# Research on nash game model for user side shared energy storage pricing

**DOI:** 10.1038/s41598-023-43254-z

**Published:** 2023-09-26

**Authors:** Weijie Qian, Chao Chen, Liwu Gong, Wei Zhang

**Affiliations:** Jiaxing Power Supply Company, State Grid Zhejiang Electric Power Co., Ltd, Jiaxing, 314000 China

**Keywords:** Energy science and technology, Engineering

## Abstract

With the continuous promotion of the energy revolution, the market-oriented reform of electricity has become the first priority in the energy field, and small-scale energy storage devices on the user side have received more and more attention. However, the disorderly management mode of user-side energy storage not only causes a waste of resources, but also brings hidden dangers to the safe operation of the power grid, such as stability, scheduling and operation, power quality and other problems. To address this issue, this paper proposes a user-side shared energy storage pricing strategy based on Nash game. Firstly, an optimal operation model is established for each participant of energy storage operators, users and grid. Secondly, a cooperative game model is established based on Nash equilibrium theory for the participants under the constraints. Finally, the optimal pricing scheme is solved by simulation analysis of the model, and the feasibility of the proposed pricing mechanism is verified. The results show that the optimal pricing scheme can achieve the purpose of peak shaving and resource saving. At the same time, it can also realize the win–win situation for all parties.

## Introduction

With the global carbon emissions increasing year by year, leading to environmental pollution and rapid climate change, it has become an important trend to vigorously develop renewable energy and realized green and low-carbon development in various industries^1,2^. As a pioneer industry to achieve carbon emission reduction, the energy industry is essential for green and low-carbon transformation. In order to improve the global ecological environment, China officially proposed the “dual carbon” goal of achieving carbon peak by 2030 and carbon neutrality by 2060 at the UN General Assembly in 2020. As the core industry in the energy sector, the digital transformation of the power industry is crucial to promote the realization of the “dual carbon” goal. Although the massive application of renewable energy in power grids has promoted the construction of clean and efficient smart grids, its strong volatility, small individual capacity and wide distribution of range have brought great challenges to the safe and stable operation of power grid^3^. However, the development of sharing economy in recent years has promoted the generation of shared energy storage, which not only smooths out the fluctuation of renewable energy but also is widely used in power system peak and frequency regulation, providing a reliable guarantee for power system supply and demand balance.

The sharing economy refers to the form in which resource owners share the right to use resources with users to earn profits and improve resource utilization efficiency. In the energy sector, the sharing economy extends to the form of shared energy storage, which separates the ownership and uses rights of energy storage^4^. Currently, there are many studies on shared energy storage by domestic and international scholars. Terlouw et al.^5^ proposed a shared energy storage deployment scenario among various users in a residential area to minimize the cost of electricity consumption and solve a multi-objective optimization problem for carbon emissions. At the same time, they used shared energy storage as an energy buffer to smooth load fluctuations and achieved energy complementarity among various users. Zhong et al.^6^ proposed a shared energy storage multi-resource allocation portfolio that linked multiple electricity users in residential areas to form a community of interests. In this way, users could purchase electrical energy resources from energy storage operators through a bidding model, thereby achieving peak-valley arbitrage. Kang Chongqing et al.^7^ studied joint investment and operation of shared energy storage by residential users. They concluded that compared to users investing in energy storage alone, the total cost of jointly investing and operating shared energy storage was reduced by 2.4%. Tian Biyuan et al.^8^ showed that the shared energy storage and demand response strategies had provided an effective guarantee for the low-carbon sustainable development of the distribution networks. They constructed a low-carbon economic dispatch model with the goal of maximizing the profit of the grid and the energy storage operator. Meanwhile, they used particle swarm optimization algorithm to solve, and verified the effectiveness of the dispatch strategy through shared energy storage regulation and load demand response. Chen Yue et al.^9^ analyzed the existing energy sharing mechanisms at domestically and internationally through both cooperative and non-cooperative games. They proposed a new concept of demand-side energy sharing, which provided an effective solution for smoothing the uncertainty of renewable energy and improving the utilization efficiency of energy storage devices.

Since there are many participating subjects in shared energy storage, the problem of benefit distribution among multiple subjects is inevitable. Each participant will face a variety of choices during the game process. According to Nash equilibrium theory, each participant will choose a certain strategy that is most beneficial to himself, regardless of which strategy other participants choose. This strategy is called the dominant strategy. If all parties in the game choose their own dominant strategies, each participant will ignore the collective interests while pursuing the maximization of individual interests, and then an equilibrium state will be reached. In this equilibrium state, as long as other participants do not change their dominant strategies, none of the participants can improve their own situation. But each participant can seek a new solution through cooperation and negotiation, and redistribute income with the goal of maximizing the overall interests, that is, Nash equilibrium. The core idea of the Nash game model is to find situations where each player cannot get a better outcome from unilaterally changing their strategy by analyzing the best strategy choices of each player. The cooperative game, as a common method to allocate interests among multiple subjects, emphasizes both individual interests and overall interests. It can achieve Nash equilibrium, thereby achieving overall optimization. Kim et al.^10^, Li et al.^11^ and Rui Tao et al.^12^ all used Nash equilibrium theory to construct a cooperative model of multi-micro-network electric energy trading. They achieved the maximization of profits in multi-micro-network cooperation by solving the model and allocating the benefits of each micro-network reasonably. Shuai et al.^13^ proposed a cooperative game-based multi-micro-network operation mechanism, and established a Nash bargaining model to rationally allocate the revenue and electricity trading volume among the micro-network. Ultimately, it achieved Pareto optimality in operating costs. Ma Tengfei et al.^14^ established a model based on Nash negotiation theory to operate wind-light-hydrogen multi-subjects in a cooperative manner. They applied the alternating direction multiplier method to solve and simulate the analysis, which significantly improved the operational efficiency of each subject and cooperative alliance. Zhang Yan et al.^15^ analyzed the optimal operation mechanism of distributed green power units and established a multi-subject cooperative game model based on Nash bargaining theory. They concluded that the participation of distributed green power units in cooperative trading of electric energy could effectively improve the operation efficiency. Gu Xin et al.^16^ analyzed the benefit distribution of multiple micro-networks in integrated energy systems, constructed a model with the objective of maximizing the overall profit of each micro-network, and allocated the benefits among the micro-network by the Nash bargaining method. Lastly, they verified through arithmetic analysis that the benefits among the micro-networks had improved compared to before the cooperation. It can be seen that Nash game has great applicability to solve the problem of multi-subject benefit distribution.

In summary, scholars at domestic and international have made some progress in the field of shared energy storage and the distribution of benefits among multiple entities. However, there are still some shortcomings. Firstly, the cost–benefit problem of shared energy storage is mainly studied, but less research is done on pricing. Secondly, it is based on the Nash game model to study the benefit distribution relationship between clean energy and multi-microgrid, which has little correlation to shared energy storage. Thirdly, research on the user-side is mainly limited to residential area users, while there is limited research on users who can configure energy storage devices themselves, such as industrial users, without considering the initiative of such users to participate in energy storage pricing. This study focuses on the above issues based on existing research and proposes a shared energy storage pricing strategy based on the Nash game model, which considers the impact of user-side distributed small energy storage on energy storage pricing. Then, this work seeks to maximize the overall benefits of each entity and the cooperative alliance through the cooperative cooperation among energy storage operators, user-side distributed small energy storage and power grid, which not only reduces the power generation cost of the power grid, but also contributes to achieve the goal of “dual carbon”.

## Analysis of the architecture and optimization model of each main body of user-side shared energy storage

### Participant structure

**Figure 1:**
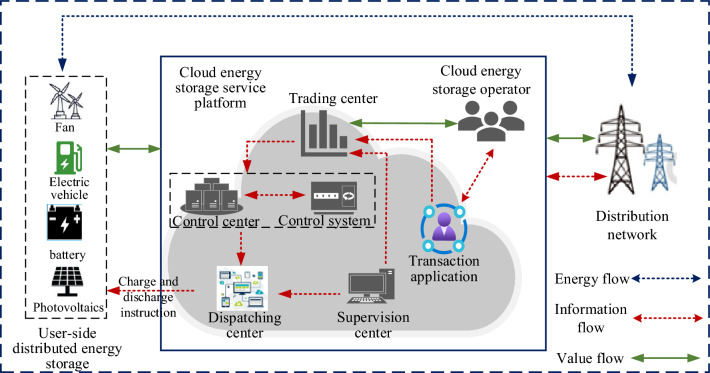
Structure of the participating subjects of the Nash game.

User-side shared energy storage participates in three categories, namely, energy storage operators, user-side distributed small energy storage and power grids. By building a cloud sharing platform, the energy storage operators collect information about the electric energy of user-side distributed energy storage and aggregate the electric energy of multiple distributed energy storage stations for unified dispatch. The power control center in the cloud energy storage service platform is the core of the whole system. Its main function is to ensure the interaction between the power control system and the cloud energy storage trading center, realize the integration of power dispatching and information technology, and complete the construction of the power dispatching basic platform. The cloud energy storage trading center is responsible for completing the transaction scheduling of online power storage units, and is supervised by the supervision center. At the same time, the transaction information and scheduling data are sent to the control center, and then the control center centrally dispatches resources and transmits them to the power demand side through the supply side of power resources, which not only ensures the security of the transaction, but also realizes the convenience of the transaction. Energy storage operators sell their electric energy to the grid at a higher price to earn revenue when the grid is undersupplied during peak periods. During periods of low electricity consumption, energy storage operators purchase electricity from the grid at a lower price for storage and use it as backup capacity to earn a peak-to-valley price differential. The user-side distributed energy storage will keep part of the stored power for self-use. At the same time, they will sell the remaining idle power to energy storage operators through the cloud energy storage service platform to earn additional revenue. In contrast to energy storage operators, the grid is able to purchase electricity at a lower price from energy storage operators during peak periods, which not only alleviates the circuit collapse caused by high circuit load during peak periods, but also ensures normal electricity consumption by users and avoids large-scale power outages. During the low period of electricity consumption, the grid sells the electricity to energy storage operators for storage, which not only achieves the effect of peak shaving and valley filling, but also reduces the cost and waste of resources, realizing the unity of economic and social benefits. The relationship between each participating entity is shown in Fig. 1.

### Optimization model for each subject's operation

#### Transaction optimization model of energy storage operators

Energy storage operators develop their own cloud dispatching platform, whose main profit *F*_*1*_ comes from the peak-valley spread revenue obtained from energy storage dispatching minus the daily operating expenses of the platform, the specific cost–benefit function is shown in Eq. (1).

1 $$\begin{array}{c}{F}_{1}={{P}_{max}\left(t\right){C}_{mAh}\left(t\right)-C}_{sop}\end{array}$$

where ${P}_{max}\left(t\right)$ is the peak electricity price in the load curve in time period *t*;${C}_{mAh}\left(t\right)$ is the electricity capacity that energy storage operators can schedule during the corresponding time period; ${C}_{sop}$ is the operating cost of the energy storage operator platform.

The operating costs of the energy storage operator platform are shown in Eq. (2).

3 $$\begin{array}{c}{C}_{sop}={C}_{GERD}+{C}_{Labor}+{C}_{ppc}\end{array}$$

where ${C}_{GERD}$, ${C}_{Labor}$, and ${C}_{ppc}$ represent the data research and development expenses, labor operation costs, and promotion expenses of the energy storage and scheduling cloud platform, respectively.

#### Optimization model of user transaction

Users store electric energy by acquiring energy storage devices, and the main profit ${F}_{i,2}$ comes from the revenue ${R}_{ESS}\left(t\right)$ obtained by selling idle electric energy other than for self-consumption minus the total cost $C\left(t\right)$. The specific cost–benefit function is shown in Eq. (3).

3 $$\begin{array}{c}{{F}_{i,2}=R}_{ESS}\left(t\right)-C\left(t\right)\end{array}$$

(1) The benefit function of user-side distributed energy storage is

4 $$\begin{array}{c}{R}_{ESS}\left(t\right)=\sum\limits_{t=1}^{T}{P}_{ESS}\left(t\right)\times {C}_{ESS}\left(t\right)\end{array}$$

where ${P}_{ESS}\left(t\right)$ is the price of electric energy sold for user-side distributed energy storage at time *t*; ${C}_{ESS}(t)$ is the storage charging capacity owned by user-side distributed energy storage at time *t*.

(2) The cost function of user-side distributed energy storage is

5 $$\begin{array}{c}C\left(t\right)={C}_{ebf}+{C}_{charge}^{t}\end{array}$$

6 $$\begin{array}{c}{C}_{ebf}=\frac{{C}_{E}}{{\varphi }_{DE}}+\frac{{C}_{P}}{D}\end{array}$$

7 $$\begin{array}{c}{C}_{charge}^{t}={P}_{min}\left(t\right){C}_{mAh}\left(t\right)\end{array}$$

where ${C}_{ebf}$ is the purchase cost of energy storage equipment; ${C}_{charge}^{t}$ is the charging cost in time period *t*; ${C}_{E}$ is the installation cost that varies with capacity; ${\varphi }_{DE}$ is the average discharge efficiency of the storage station; ${C}_{P}$ is the installation cost that varies with power; *D* is the average discharge duration at rated power; ${P}_{min}\left(t\right)$ refers to the low valley electricity price of the load curve during period *t*; ${C}_{mAh}\left(t\right)$ is the battery capacity possessed during the corresponding time period.

#### Power grid transaction optimization model

The power grid cost–benefit function is

8 $$\begin{array}{c}{F}_{3}=P\left(t\right){G}_{mAh}\left(t\right)-\left({N}_{pce}{C}_{pce}+{G}_{mAh}\left(t\right){C}_{sup}\right)\end{array}$$

9 $$\begin{array}{c}{C}_{pce}=\sum\limits_{i=1}^{n}{N}_{pce}\left(i\right){C}_{pce}\left(i\right)/{N}_{pce}\left(All\right)\end{array}$$

10 $$\begin{array}{c}{C}_{sup}=\left({C}_{pro}+{C}_{op}+{C}_{ma}+{C}_{fin}\right)/{G}_{mAh}\left(All\right)\end{array}$$

where Profit *F*_*3*_ = unit price of electricity sale × selling electricity—(purchasing electricity × unit electricity purchase cost + electricity sold × unit power supply cost); ${N}_{pce}\left(i\right)$, ${C}_{pce}(i)$ and ${N}_{pce}(All)$ refer to the purchase of electricity in different categories, the purchase unit price of electricity in different categories, and the total purchase of electricity; ${C}_{pro}$, ${C}_{op}$, ${C}_{ma}$, ${C}_{fin}$ and ${G}_{mAh}\left(All\right)$ represent production costs, operating expenses, management expenses, financial expenses, and total sold electricity, respectively.

## Research on the Nash game model of user-side shared energy storage

The user-side shared energy storage Nash game model based on Nash equilibrium theory aims at the optimal benefit of each participant and considers the constraints such as supply and demand equilibrium, so as to achieve the overall optimal and obtain the best strategy choice. User-side shared energy storage is composed of interconnection and mutual benefit of adjacent energy storage devices in the same area, so the power loss in the power interaction process can be ignored^17^.

### Operating mechanism of the cooperative game model

Without considering the energy loss cost of power interaction between the partners of the alliance, all participants hope to maximize their own interests in the process of cooperative operation, that is, the overall benefit is optimal in the state of cooperation. The Nash equilibrium model is shown below.

11 $$\begin{array}{c}\mathrm{max}{[(F}_{1}-{F}_{1}^{0})\prod\limits_{i=1}^{x}{(F}_{i,2}-{F}_{i,2}^{0})({F}_{3}-{F}_{3}^{0})]\end{array}$$

12 $$\begin{array}{c}{F}_{1}\ge {F}_{1}^{0};{F}_{i,2}\ge {F}_{i,2}^{0};{F}_{3}\ge {F}_{3}^{0}\end{array}$$

13 $$\begin{array}{c}\mathrm{max}{(F}_{1}+\sum\limits_{i=1}^{x}{\overline{F} }_{i,2}+{F}_{3})\end{array}$$

14 $$\begin{array}{c}{\delta }_{i,ESS}=\frac{F\left(R\right)-F(R-\left\{i\right\})}{F\left(R\right)}\end{array}$$

15 $$\begin{array}{c}max[\mathrm{ln}\left({F}_{1}^{*}+{N}_{pce}{C}_{pce}-{R}_{ESS}\left(t\right)-{F}_{1}^{0}\right)+\sum\limits_{i=1}^{x}{\delta }_{i,ESS}ln({\overline{F} }_{i,2}^{*}+{R}_{ESS}\left(t\right)-{F}_{i,2}^{0})\\ +\mathrm{ln}\left({F}_{3}^{*}-{N}_{pce}{C}_{pce}-{F}_{3}^{0}\right)]\end{array}$$

16 $$\begin{array}{c}{F}_{1}^{*}+{N}_{pce}{C}_{pce}-{R}_{ESS}\left(t\right)-{F}_{1}^{0}\ge 0\end{array}$$

17 $$\begin{array}{c}{\overline{F} }_{i,2}^{*}+{R}_{ESS}\left(t\right)-{F}_{i,2}^{0}\ge 0\end{array}$$

18 $$\begin{array}{c}{F}_{3}^{*}-{N}_{pce}{C}_{pce}-{F}_{3}^{0}\ge 0\end{array}$$

where ${F}_{1}^{0}$, ${F}_{i,2}^{0}$ and ${F}_{3}^{0}$ are the optimal operating benefits of energy storage operators, distributed energy storage on each user side and power grid in the absence of cooperation, and are also the breakdown points of negotiations; ${\overline{F} }_{i,2}$ is the average of ${F}_{i,2}$; ${\delta }_{i,ESS}$ is the bargaining power of distributed energy storage on the *i* user side; $F\left(R\right)$ is the total revenue of cooperative operation; $F(R-\left\{i\right\})$ is the total revenue for the *i*-user-side distributed energy storage without participating in cooperative operation; ${F}_{1}^{*}$, ${\overline{F} }_{i,2}^{*}$ and ${F}_{3}^{*}$ are the solutions of formula (13) respectively. The constraints of this model are as follows:

(1) Balance constraint of supply and demand

19 $$\begin{array}{c}{P}_{t}^{sup}={P}_{t}^{dem}\end{array}$$

where ${P}_{t}^{sup}$ is the electricity supply during period *t*; ${P}_{t}^{dem}$ is the electricity demand during period *t*.

(2) Charging number constraint

20 $$\begin{array}{c}{C}_{edc}={C}_{sto}\times \left(\frac{1-r}{{T}_{discharge}^{charge}}\times {U}_{times}\right)\end{array}$$

where ${C}_{edc}$ is the depreciation cost of energy storage equipment; ${C}_{sto}$ is the construction cost of energy storage equipment; *r* is the net residual value rate of energy storage equipment; ${T}_{discharge}^{charge}$ is the expected total number of charges and discharges for the energy storage equipment; ${U}_{times}$ is the numbers of charges and discharges that have been used.

### Cooperative game model solution

**Figure 2:**
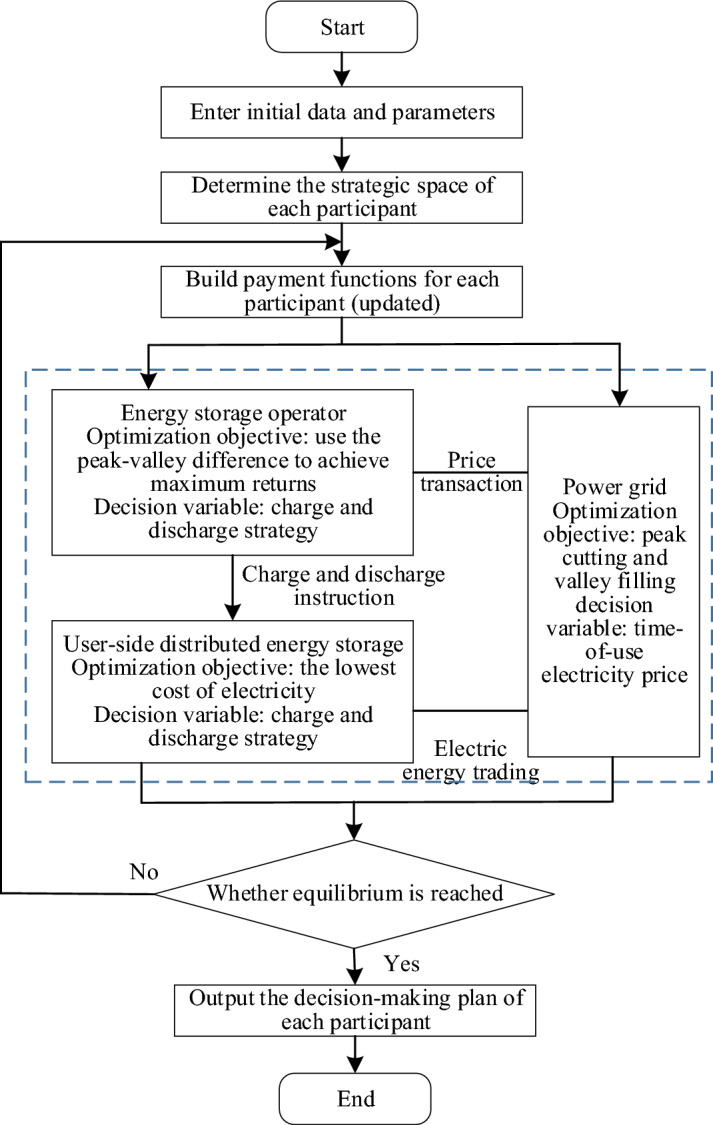
Flow chart of the Nash equilibrium model of user-side shared energy storage.

The Nash equilibrium model is essentially a non-convex nonlinear integer programming problem coupled with multiple variables. It is a model constructed with the objective of maximizing the benefits of energy storage operators, power grid, and user-side distributed energy storage. As shown in Fig. 2, the specific solving process is as follows.

*Step 1* Set the initial data and parameters for each participant.

*Step 2* The data are processed to determine the strategic space of each participant. The continuous variables such as the output of each generator set and the charge and discharge power of the energy storage device are divided into several intervals. The length of which is

21 $$\begin{array}{c}\Delta {L}_{k}=\frac{{L}_{k}^{max}-{L}_{k}^{min}}{{N}_{k}}\end{array}$$

where $\Delta {L}_{k}$ is the length of each interval; ${L}_{k}^{max}$ and ${L}_{k}^{min}$ are the maximum and minimum output values of the k device, respectively; ${N}_{k}$ is the number of intervals.

*Step 3* Construct the payment function of each participant and calculate the utility function value of each player. Analyze the optimal response strategies of each participant when faced with the choices of other participants.

*Step 4* Determine whether Nash equilibrium is reached. When the utility function values of each game player get the same solution in two adjacent iterations, the iteration will be stopped. Otherwise, the program returns to the third step, modifies the payment function, optimizes the strategy combination, and iterates again.

*Step 5* Output the decision-making scheme of each participant, that is, the optimal solution.

## Example and analysis

### Example parameter setting

To verify the effectiveness of the Nash equilibrium model of user-side shared energy storage, the actual operation data of different user-side distributes energy storage in an industrial park in Northeast China is selected as the initial value of the example analysis, as shown in Table 1.

**Table 1:** Setting of distributed energy storage parameters on the user-side.

Energy storage parameter setting	
Rated voltage	380 V
Rated capacity	265 kW
Charging parameters	420 V 300 A
Charger power	126 kW
Output rating	58.2 kW

**Figure 3:**
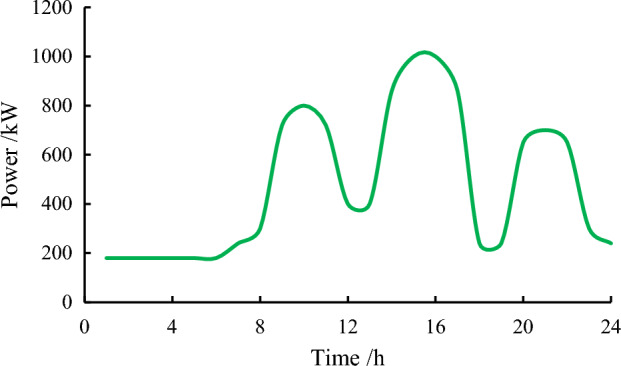
Daily load curve of electricity consumption in industrial parks.

Since the electricity load in summer in this region is significantly higher than that in other seasons and has greater adjustment potential, this study analyzes the typical daily electricity load in summer. The model takes 24 h as a power consumption cycle and takes 15 min as a sampling interval. The typical daily power load curve consists of 96 discrete points, as shown in Fig. 3.

Due to the different electricity consumption characteristics of users, the electricity load curve fluctuates greatly. Therefore, the electricity prices of the industrial parks where each user is located are divided into three levels: peak hour electricity price, regular hour electricity price, and valley hour electricity price according to the peak, flat peak, and low valley periods of electricity consumption. The ratio of the three is 3:2:1, and the specific parameter settings are shown in Table 2.

**Table 2:** Initial electricity price at different time periods.

Electricity price category	Period of time	Electricity price (CNY /kWh)
Peak hour electricity price	9:00–12:00 14:00–18:00 20:00–22:00	1.308
Ordinary electricity price	7:00–9:00 12:00–14:00 18:00–20:00 22:00–24:00	0.872
Valley hour electricity price	0:00–7:00	0.436

### Analysis and discussion of algorithm results

The Nash equilibrium model was established by Matlab2021a-Yalmip and solved by invoking CPLEX optimization software. On the one hand, the user-side distributed energy storage device can store electricity through renewable energy sources such as scenery during the day; On the other hand, the energy storage device can buy energy storage during the low price of electricity at night. User-side distributed energy storage adopts the mode of self-use and surplus power online, which not only saves electricity cost, but also can earn income through the peak-valley price difference. Therefore, in order to make all the surplus electricity go online and avoid waste of resources, the electricity price sold by the user-side distributed energy storage needs to be lower than the initial electricity price of the grid, which is highly attractive to buyers. The three optimized electricity price combinations determined based on the Nash game are all located between the repurchase electricity price and the sale electricity price in the distribution network, as shown in Table 3.

**Table 3:** Model optimization of electricity price combination.

	Peak hour electricity price (CNY/kWh)	Ordinary electricity price (CNY/kWh)	Valley hour electricity price (CNY/kWh)
Option 1	1.279	0.859	0.423
Option 2	1.235	0.843	0.418
Option 3	1.174	0.826	0.401

**Figure 4:**
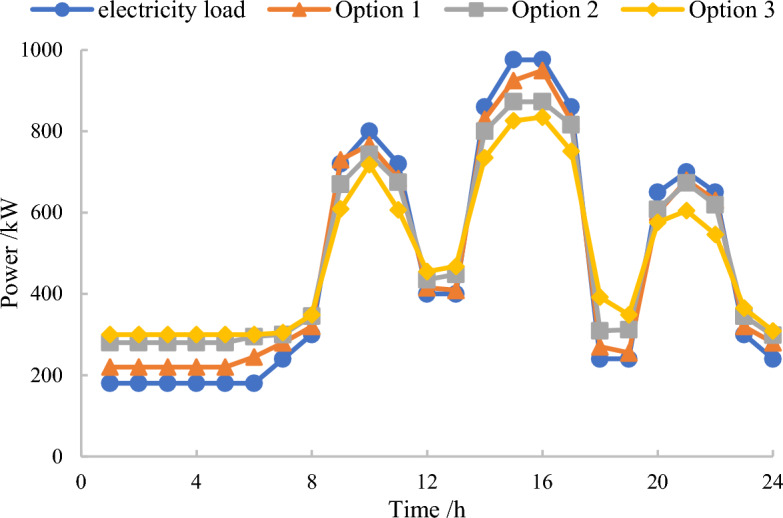
Load curve of each scheme under cooperative operation.

In the traded competitive market, energy storage operators will forecast the power generation of distributed power sources. And user-side distributed energy storage will also publish its own output information on the cloud energy storage service platform, including phased electricity prices, available power supply, etc. Real time scheduling within the system can be achieved through negotiations between energy storage operators and the power grid. A comparison of load curves in response to demand before and after cooperative operation is shown in Fig. 4.

The response results indicate that energy storage operators guide user-side distributed small energy storage to schedule backup energy storage from 9:00 a.m. to 12:00 a.m. and from 14:00 p.m. to 18:00 p.m. to participate in peak load reduction. Meanwhile, they guide user-side distributed small energy storage to conduct unified charging behavior as much as possible during the low load period from 1:00 p.m. to 6:00 p.m. It can increase the backup capacity, reducing the electricity consumption during peak hours by 100–300 kW, and increasing the electricity consumption during peak and normal periods by about 100 kW and 60 kW, respectively.

From the three load curves after scheduling, it can be seen that regardless of the specific pricing strategy, user-side distributed small energy storage is charged uniformly during low load periods and periods with high wind and solar output, and discharged uniformly during peak electricity consumption periods in the morning, afternoon, and evening, which to some certain extent plays a role in “peak shaving and valley filling” for the original load. However, due to the balance of supply and demand in the electricity trading market and the related cost and revenue constraints, a small amount of energy storage is wasted from 12:00 a.m. to 14:00 p.m. and 18:00 p.m. to 19:00 p.m.

**Figure 5:**
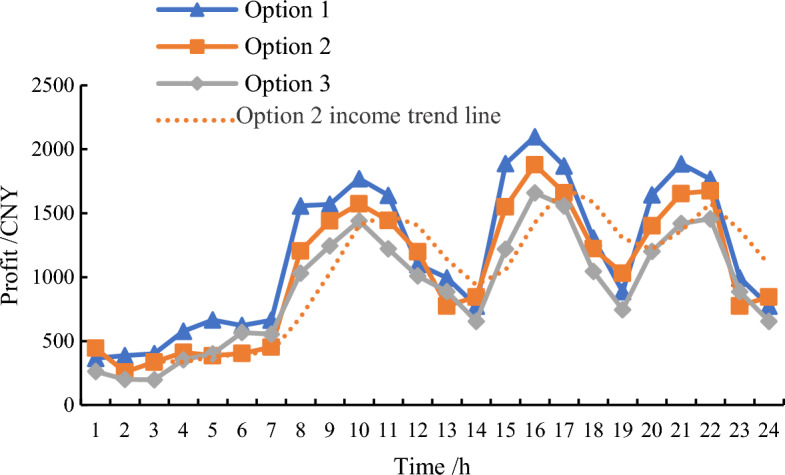
Revenue curves of each scheme under cooperative operation.

Based on the daily electricity load of each scheme under cooperative operation state, the revenue curve of shared energy storage on the user-side is solved, as shown in Fig. 5.

Without considering the energy loss cost of electricity interaction between alliance entities, the calculation results show that: Under the first model, the energy storage operator sells power to the grid at a higher price. At this point, the energy storage operator and the user-side of the small energy storage revenue is larger. But for the grid, the cost of power purchase is higher and the load curve “peak and valley reduction” is the least obvious, the comprehensive benefits are minimal, and the desire to purchase energy storage is weak.

In the second mode, the peak hour tariff is 1.235 CNY/kWh, the weekday tariff is 0.843 CNY/kWh, and the valley tariff is 0.418 CNY/kWh. The negotiation breakdown point is obtained by the intersection of the trend line and the actual return curve of Option 2, which are located at 11:00 a.m. and 17:00 p.m. in the peak hours respectively. The return at 11:00 a.m. is 1467 CNY, and the return at 17:00 p.m. is 1660 CNY. So, 17 o'clock is the optimal solution reached by the tripartite cooperation under the Nash equilibrium model.

In the third mode, the electricity price is the lowest in each period, so the cost of purchasing power from energy storage operators is lower and the income is higher. For power grid, the effect of peak cutting and valley filling is the most significant. While for the energy storage operator and small storage on the customer side, the price of electricity sold at this time is lower, the revenue is smaller, and the willingness to cooperate is weaker.

By comparing the three tariff combinations, it can be seen that Option 2 has moderate tariffs for each time period, balanced revenue for all three parties, and a smoother load curve. Which not only makes more profit for the customer-side small storage that sells electricity, but also saves more power purchase cost for the grid that buys electricity. Compared with the traditional transaction mode, all parties have obtained higher returns and maximized the overall benefits. The cooperative alliance can be maintained. Therefore, the second mode is the best combination of electricity pricing solutions that have been achieved through cooperation among all parties.

## Conclusion

In this paper, three pricing schemes based on Nash game model are proposed to maximize the profit distribution among multiple entities on the basis of the cooperative operation of each participant of shared energy storage. The models are verified according to three different electricity price schemes, and the optimal scheme is solved. The analysis results show that:

1. The user-side distributed small energy storage is dispatched through the cloud energy storage service platform. It not only reduces the user's power purchase during the peak period of electricity consumption, which has a certain effect on flattening the load curve, but also achieves the role of “peak shaving and valley filling”. It also increases additional income for users and reduces the cost of living.
2. The energy storage operator negotiates with the grid on behalf of users, sets reasonable pricing for purchase and sale, and flexibly dispatches electricity through multiple small distributed energy storage, which provides a reliable guarantee for the balance between supply and demand of electricity. In addition, the energy storage operator can obtain maximum revenue through pricing strategy under the condition of ensuring feasible revenue for three parties.
3. The power grid purchases and sells electricity at different times through the cloud energy storage service platform, which reduces the waste of electricity while maintaining the safe and stable operation of the power grid. It is conducive to reducing the cost of power generation, improving the consumption of scenic power generation, responding to the national call of “energy saving and emission reduction”, and realizing the win–win situation of economic and social benefits.

## Data Availability

The data that support the findings of this study are available from [Power companies in China] but restrictions apply to the availability of these data, which were used under license for the current study, and so are not publicly available. Data are however available from the authors upon reasonable request and with permission of [Power companies in China]. If you need to obtain data, please contact Weijie Qian. z2602087886@163.com.
